# The serotonin 2A receptor agonist 25CN-NBOH increases murine heart rate and neck-arterial blood flow in a temperature-dependent manner

**DOI:** 10.1177/0269881120903465

**Published:** 2020-02-12

**Authors:** Tobias Buchborn, Taylor Lyons, Chenchen Song, Amanda Feilding, Thomas Knöpfel

**Affiliations:** 1 Laboratory for Neuronal Circuit Dynamics, Department of Medicine, Imperial College, London, UK; 2 Centre for Neurotechnology, Institute of Biomedical Engineering, Imperial College, London, UK; 3 Centre for Psychedelic Research, Department of Medicine, Imperial College, London, UK; 4 The Beckley Foundation, Oxford, UK

**Keywords:** 25CN-NBOH, bradypnea, carotid artery, haemodynamics, hypertension, psychedelic, selective 5-HT_2A_R agonist, tachycardia, thermoregulation, vasoactive

## Abstract

**Background:**

Serotonin 2A receptors, the molecular target of psychedelics, are expressed by neuronal and vascular cells, both of which might contribute to brain haemodynamic characteristics for the psychedelic state.

**Aim:**

Aiming for a systemic understanding of psychedelic vasoactivity, here we investigated the effect of N-(2-hydroxybenzyl)-2,5-dimethoxy-4-cyanophenylethylamine – a new-generation agonist with superior serotonin 2A receptor selectivity – on brain-supplying neck-arterial blood flow.

**Methods:**

We recorded core body temperature and employed non-invasive, collar-sensor based pulse oximetry in anesthetised mice to extract parameters of local blood perfusion, oxygen saturation, heart and respiration rate. Hypothesising an overlap between serotonergic pulse- and thermoregulation, recordings were done under physiological and elevated pad temperatures.

**Results:**

N-(2-hydroxybenzyl)-2,5-dimethoxy-4-cyanophenylethylamine (1.5 mg/kg, subcutaneous) significantly increased the frequency of heart beats accompanied by a slight elevation of neck-arterial blood flow. Increasing the animal-supporting heat-pad temperature from 37°C to 41°C enhanced the drug’s effect on blood flow while counteracting tachycardia. Additionally, N-(2-hydroxybenzyl)-2,5-dimethoxy-4-cyanophenylethylamine promoted bradypnea, which, like tachycardia, quickly reversed at the elevated pad temperature. The interrelatedness of N-(2-hydroxybenzyl)-2,5-dimethoxy-4-cyanophenylethylamine’s respiro-cardiovascular effects and thermoregulation was further corroborated by the drug selectively increasing the core body temperature at the elevated pad temperature. Arterial oxygen saturation was not affected by N-(2-hydroxybenzyl)-2,5-dimethoxy-4-cyanophenylethylamine at either temperature.

**Conclusions:**

Our findings imply that selective serotonin 2A receptor activation modulates systemic cardiovascular functioning in orchestration with thermoregulation and with immediate relevance to brain-imminent neck (most likely carotid) arteries. As carotid branching is a critical last hub to channel cardiovascular output to or away from the brain, our results might have implications for the brain haemodynamics associated with psychedelia.

## Introduction

Psychedelics (serotonergic hallucinogens) are psychoactive drugs whose effects show strong manifestation in human psychological functioning and in various somatic alterations across mammals. Lysergic acid diethylamide (LSD) *in humano*, for instance, has been shown to induce pupil dilatation, patellar hyperreflexia, temperature dysregulation, as well as increased breath rate, heart rate and blood pressure (Isbell, 1959; Schmid et al., 2015). In animals, stereotypical movements, including head twitches and wet dog shakes (e.g. Buchborn et al., 2015; Corne and Pickering, 1967) as well as other motor symptoms referred to as *serotonin syndrome*, have been described (Sloviter et al., 1980). Beyond neuro-summative electroencephalography (EEG) and magnetoencephalography (MEG) recordings, our knowledge of the neurophysiological correlates of the human psychedelic brain state largely feeds from blood-oxygen-level-dependent functional magnetic resonance imaging (BOLD-fMRI) and positron-emission tomography (PET)based research. As the given methods indirectly measure neuronal activity by extraction of blood-flow related parameters, the vasoactivity intrinsic to psychedelics deserves particular experimental scrutiny when interpreting such correlates. Psychedelics are thought to primarily mediate their psychedelic effect by activating serotonin 2A receptors (5-HT_2A_Rs) (Kraehenmann et al., 2017; Preller et al., 2017), with glutamatergic pyramidal cells within the cerebral cortex proposed as one of the key sites of their action (Marek, 2017; Muthukumaraswamy et al., 2013; Nichols, 2016; Vollenweider and Kometer, 2010). 5-HT_2A_Rs – apart from neuronal expression in the brain (see the G-protein coupled receptor [GPCR] database; Andrade and Weber, 2010; Regard et al., 2008) – are widely expressed across the vascular system (Cohen, 1988; Ullmer et al., 1995) and there is ongoing controversy as to how a direct interaction between psychedelics and the vessels might contribute to the overall corticodynamics associated with the psychedelic state (Lewis et al., 2017; Muller et al., 2018). Psychedelics are likely to interfere with cerebral blood flow by targeting 5-HT receptors within the brain’s microcirculation itself (Cohen et al., 1996). Despite autoregulatory shielding of brain blood flow from the caprioles of the periphery (Yang and Liu, 2017), heart rate and systemic blood pressure also have a clear effect on cerebral haemodynamics (Kalisch et al., 2005; Ma et al., 2016; Terem et al., 2018). BOLD-fMRI-based brain functioning studies, thought to mainly reflect neuronal activity, might therefore partially be confounded by local and/or cardiovascular bottom-up effects (e.g. de Munck et al., 2008; van’t Ent et al., 2014). Although there is general effort to control for these complex interactions in brain imaging studies on psychedelics (Carhart-Harris et al., 2012; Müller et al., 2018), it appears essential to learn how 5-HT_2A_R activation influences the brain’s supply of blood when disentangling neuro- and haemodynamic mechanisms involved in the psychedelic brain state. Cardiovascular receptors of the 5-HT_2_ family (5-HT_2_Rs) have been subject to extensive research (Kermorgant et al., 2018; rev. McCall and Clement, 1994; Nagatomo et al., 2004; Ramage, 2001), yet our understanding of how they regulate systemic circulation has largely been occluded by the unavailability of selective agonists. The phenylaminergic psychedelics 2,5-dimethoxy-4-iodoamphetamine (DOI) and 2,5-dimethoxy-4-methylamphetamine (DOM) for instance, which so far have been the state-of-the-art drugs for delineating 5-HT_2_R-specific cardiovascular functioning, do not seem to well discriminate 5-HT_2A_Rs from 5-HT_2B_ and/or 5-HT_2C_ receptors (e.g. Porter et al., 1999; Sanders-Bush et al., 1988). Also, it has been suggested they might have confounded affinity for adrenergic receptors (Ray, 2010), making an appraisal of 5-HT_2A_R specific vasoactive effects difficult.

Here, we used N-(2-hydroxybenzyl)-2,5-dimethoxy-4-cyanophenylethylamine (25CN-NBOH), a recently developed selective 5-HT_2A_R agonist with 100-fold preference for 5-HT_2A_Rs over a plethora of non-5-HT_2_R targets (Jensen et al., 2017). We tested 25-CN-NBOH with regard to its effects on heart rate, blood flow, blood oxygenation and respiratory rate in mice, monitored by non-invasive pulse oximetry drawn from the animals’ brain-imminent neck arteries. In both animals and humans, psychedelics are known to strongly exalt emotions (e.g. Adams and Geyer, 1985; Katz et al., 1968). To avoid cardiovascular effects associated with emotional arousal (Hoyt et al., 2007; Prkachin et al., 1999), experiments were performed under anaesthesia. Furthermore, as 5-HT_2A_R-related vasoconstriction is thought to be a main effector site of serotonergic thermoregulation (e.g. Ootsuka et al., 2004), we expected the environmental temperature to be critical for psychedelic haemodynamics. We therefore explored the drug’s cardiovascular effects by varying the temperature of the heat-pad supporting the animals.

## Methods

### Animals

All experimental procedures performed at Imperial College London were in accordance with the United Kingdom Animal Scientific Procedures Act (1986) under Home Office Personal and Project licences (I5B5A6029, IA615553C; PPL 70/7818), following appropriate ethical review. Adult mice of both sexes and with mixed (non-inbred) genetic background (stock of mainly C57BL/6JxB6CBAF1 background; *N* = 41) were bred in-house at the Central Biomedical Services of Imperial College London. They were housed in individually ventilated cages with a 12:12 h light/dark cycle and maintained at an ambient temperature of 21 ± 2°C at 55 ± 10% humidity. Mice were provided with standard rodent-chow pellets (Special Diet Services, #RM1) and water *ad libitum*.

### Drugs

The 25CN-NBOH hydrochloride (a kind gift from Jesper L. Kristensen, University of Copenhagen) was dissolved in isotonic saline and applied subcutaneously (<10 mL/kg). The dose used (1.5 mg/kg) was found optimal for a 5-HT_2A_R-specific effect, as determined by a dose-response curve for 25CN-NBOH-induced head twitches (Buchborn et al., 2018). Experiments were performed under general anaesthesia using isoflurane (IsoFlo; Zoetis, UK) delivered in 100% oxygen, as detailed below.

### Pulse oximetry and core body temperature measurements

**Figure 1.**
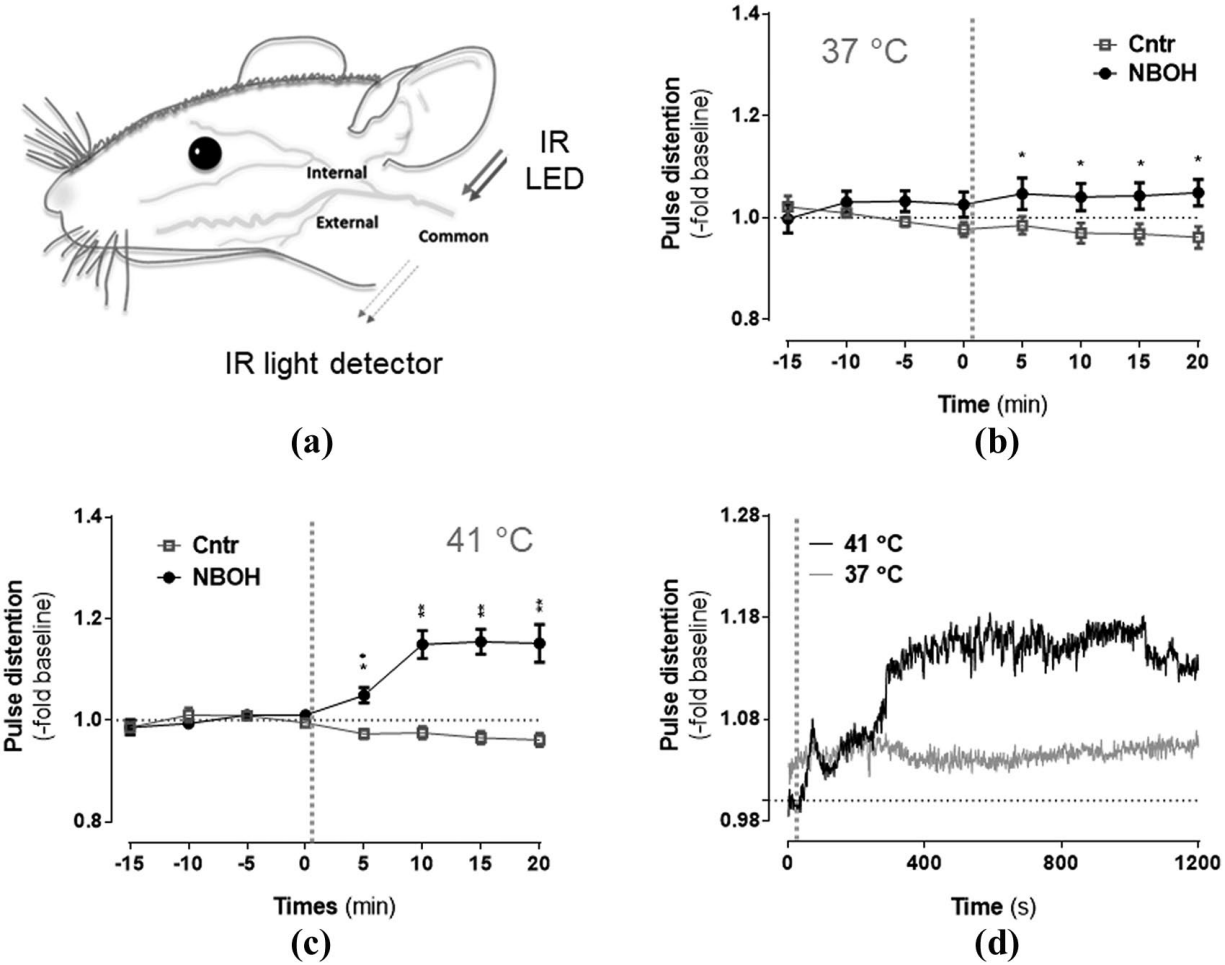
Effect of N-(2-hydroxybenzyl)-2,5-dimethoxy-4-cyanophenylethylamine (25CN-NBOH) (1.5 mg/kg, subcutaneous) on arterial distension of isoflurane-anaesthetised mice. (a) Schematic depiction of the major carotid branches of the mouse with area of neck-arterial pulse oximetry indicated. Infrared (IR) light from a light emitting diode (LED) is picked up by an IR light detector after passing the perfused tissue. Recordings at (b) physiological and (c) elevated pad temperatures. Mean ± SEM (fold change of baseline, μm); *n* = 8–14 per group. Repeated measures analysis of variance (ANOVA) comparison to saline, ^*^p ⩽ 0.05 and ^**^p ⩽ 0.01. (d) Temperature-dependency of 25CN-NBOH’s effect on pulse distension, depicted in 1 Hz resolution. Vertical dotted lines in (b–d) indicate time of drug injection.

Pulse oximetry was performed in anaesthetised mice using the rodent-specific MouseOx Plus collar-sensor system (STARR Life Sciences Corp., Oakmont, PA). Anaesthesia was induced in an anaesthesia chamber (VetTech Solutions, Cheshire, UK) at 2–2.5% isoflurane and, following the loss of the righting reflex, maintained via two-port mask supply at 1.5–2.0% concentration. Body temperature was maintained by a feedback-loop controlled heat-pad (TR-200 Fine Science Tools, USA), which drew input from a thermosensor attached to the heat-pad’s surface, controlling for physiological and elevated (37°C and 41°C) pad temperatures, respectively. The collar-sensor was placed around the shaved necks of the animals to read oxygen saturation (percentage of functional arterial haemoglobin), heart rate (beats per minute (bpm)), respiration rate (breaths per minute (brpm)) and pulse distention of carotid arteries (Figure 1(a)). Pulse distention reflects changes in light absorption by haemoglobin and via Beer’s law provides the length of the optical path through the blood (Hete et al., 2013). All parameters were calculated by the MouseOx system and read out at 1 Hz, with a 20-minute baseline period, followed by injection of 25CN-NBOH and saline, and a 20-minute post-injection period. Core body temperature was monitored via a digital thermometer and rectal probe and registered at 2-minute intervals.

### Statistics

Pulse oximetry 1 Hz readouts – as aggregated in 5-minute bins – and core body temperature measurements were analysed via SPSS-implemented three-factorial repeated measures analysis of variance (ANOVA) (with *group* and *pad temperature* as between-subject factors and *time* as within-subject factor) and analysed for main effects, interaction and contrast. Significant interactions were followed by Bonferroni-corrected multiple comparisons. For cross-parameter comparability, results were normalised to their respective baseline average and depicted as fold of baseline.

## Results

### Pulse distention

For isoflurane-anaesthetised control animals at both 37°C and 41°C pad temperatures (*n* = 8 and 14, respectively), pulse distention remained relatively stable throughout the 40-minute recording period. This contrasts with 25CN-NBOH-treated mice (Figure 1; repeated measures ANOVA, significant differences as a function of *time* (*F*(2.52, 93.27) = 3.51, *p* = 0.001), *group* (*F*(1, 37) = 3.51, *p* ⩽ 0.001), *time* × *group* (*F*(2.52, 16.37) = 16.37, *p* ⩽ 0.001), as well as *time* × *group* × *temperature* (*F*(2.52, 93.27) = 4.99, *p* = 0.005): Bonferroni-corrected post-hoc analysis revealed significant group differences from 25CN-NBOH treated animals (*n* = 9 and 10, respectively) at any of the four post-injection measurements for both temperature conditions (control vs. NBOH (mean ± SEM, fold change) for 37°C: 0.98 ± 0.017 vs. 1.05 ± 0.03 (5 min), *p* = 0.021; 0.97 ± 0.019 vs. 1.04 ± 0.026 (10 min), *p* = 0.036; 0.96 ± 0.019 vs. 1.04 ± 0.026 (15 min), *p* = .022; 0.96 ± 0.021 vs. 1.049 ± 0.026 (20 min), *p*=0.028; for 41°C: 0.97 ± 0.01 vs. 1.05 ± 0.015 (5 min), *p* = 0.005; 0.97 ± 0.012 vs. 1.15 ± 0.027 (10 min), *p* ⩽ 0.001; 0.96 ± 0.012 vs. 1.15 ± 0.024 (15 min), *p* ⩽ 0.001; 0.96 ± 0.013 vs. 1.15 ± 0.037 (20 min), *p* ⩽ 0.001). At an increased pad temperature, the 25CN-NBOH-induced increase in arterial distension was much more pronounced (NBOH 37°C vs. 41°C: *p* = 0.026 (5 min); *p* = 0.031 (10 min); *p* = 0.029 (15 min); *p* = 0.036 (20 min)). For the saline-treated control mice, no such temperature effect could be found (control 37°C vs. 41°C: *p* = 0.93 (5 min); *p* = 0.85 (10 min); *p* = 0.92 (15 min); *p* = 0.99 (20 min)). Mean absolute values of arterial distension for the pre- versus post-injection intervals (given in μm) are presented in Tables 1 and 2.

**Table 1.** Effect of 25CN-NBOH (1.5 mg/kg, s.c.) on autonomic functioning of isoflurane-anaesthetised mice at the 37°C pad temperature. Mean (SEM) absolute values of 20-minute baseline versus 20-minute post-injection period. *N* = 6–14 per group.

37°C	Saline		NBOH	
	Baseline	Post-injection	Baseline	Post-injection
**Pulse distention** (μm)	478 (36)	464 (38)	497 (24)	508 (25)
**Heart rate** (bpm)	459 (19)	492 (24)	430 (17)	511 (15)
**Respiration rate** (brpm)	69 (6)	63 (7)	76 (5)	59 (15)
**Oxygenation** (%)	99 (0.1)	99 (0.1)	99 (0.1)	99 (0.1)
**Temperature** (°C)	37 (0.2)	37 (0.2)	37 (0.2)	37 (0.2)

25CN-NBOH: N-(2-hydroxybenzyl)-2,5-dimethoxy-4-cyanophenylethylamine; bpm: beats per minute; brpm: breaths per minute.

**Table 2.** Effect of 25CN-NBOH (1.5 mg/kg, s.c.) on autonomic functioning of isoflurane-anaesthetised mice at the 41°C pad temperature. Mean (SEM) absolute values of 20-minute baseline versus 20-minute post-injection period. *N* = 6–14 per group.

41°C	Saline		NBOH	
	Baseline	Post-injection	Baseline	Post-injection
**Pulse distention** (μm)	484 (37)	468 (35)	462 (30)	517 (30)
**Heart rate** (bpm)	466 (16)	509 (16)	474 (18)	523 (15)
**Respiration rate** (brpm)	81 (7)	70 (7)	84 (10)	67 (8)
**Oxygenation** (%)	99 (0.06)	99 (0.06)	99 (0.07)	99 (0.07)
**Temperature** (°C)	37 (0.2)	37 (0.2)	37 (0.2)	38 (0.3)

25CN-NBOH: N-(2-hydroxybenzyl)-2,5-dimethoxy-4-cyanophenylethylamine; bpm: beats per minute; brpm: breaths per minute.

### Heart rate

**Figure 2.**
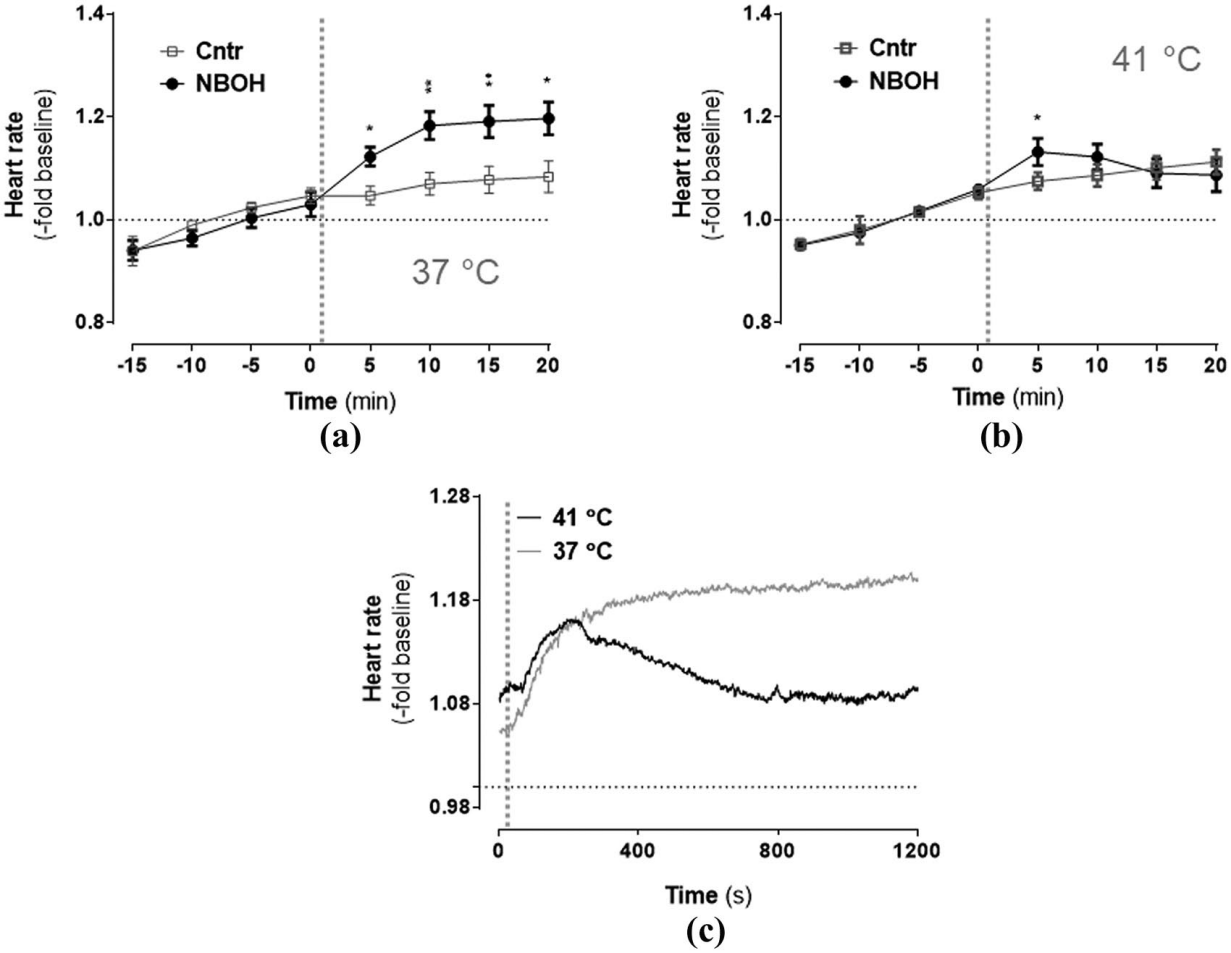
Effect of N-(2-hydroxybenzyl)-2,5-dimethoxy-4-cyanophenylethylamine (25CN-NBOH) (1.5 mg/kg, subcutaneous) on the heart rate of isoflurane-anaesthetised mice at (a) physiological and (b) elevated pad temperatures. Mean ± SEM (fold change of baseline bpm); *n* = 8–14 per group. Repeated measures analysis of variance (ANOVA) comparison to saline, ^*^p ⩽ 0.05 and ^**^p ⩽ 0.01. (c) Temperature-dependency of 25CN-NBOH’s effect on heart rate, depicted in 1 Hz resolution. Vertical dotted lines indicate time of drug injection.

Isoflurane-anesthetised mice overall exhibited a tendency for an increase of heart rate over time (Figure 2; repeated measures ANOVA, significant main effect *time: F*(1.77, 65.40) = 65.76, *p* ⩽ 0.001). Furthermore, a significant *time × group* interaction (*F*(1.77, 65.40) = 4.59, *p* ⩽ 0.001) and a significant *time* × *treatment* × *temperature* interaction (*F*(1.77, 65.40) = 3.93, *p* = 0.029) indicated the temporal changes in heart rate differed between both groups in a temperature-dependent manner. Bonferroni-corrected post-hoc comparisons revealed the significant *time* × *group* interaction was due to an 25CN-NBOH-induced tachycardia at all four post-injection measurements for the 37°C condition (control vs. NBOH (mean ± SEM, -fold change): 1.05 ± 0.018 vs. 1.12 ± 0.018 (5 min), *p* = 0.041; 1.07 ± 0.02 vs. 1.18 ± 0.027 (10 min), *p* ⩽ 0.001; 1.08 ± 0.026 vs. 1.19 ± 0.031 (15 min), *p* = 0.01; 1.08 ± 0.03 vs. 1.19 ± 0.032 (20 min), *p* = 0.016) and at the first post-injection measurement for the 41°C condition (control vs. NBOH (mean ± SEM, fold change): 1.07 ± 0.016 vs. 1.13 ± 0.026 (5 min), *p* = 0.044). Accordingly, temperature-dependent heart rate differences in 25CN-NBOH-treated animals could be detected at the 15- and 20-minute measurements and as a trend for the 10-minute measurement (NBOH 37°C vs. 41°C: *p* = 0.095 (10 min); *p* = 0.015; 40 min (15 min): *p* = 0.029; *p* = 0.014 (20 min)). Mean absolute values for the pre- versus post-injection intervals (given in bpm) are shown in Tables 1 and 2.

### Respiration rate

**Figure 3.**
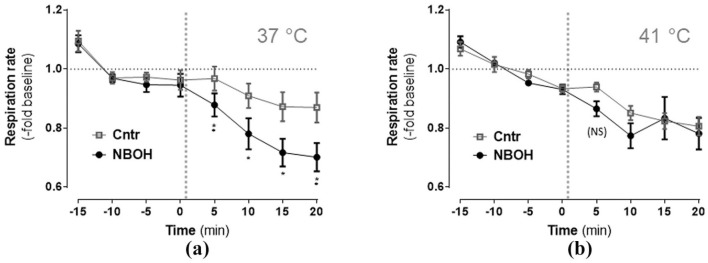
Effect of N-(2-hydroxybenzyl)-2,5-dimethoxy-4-cyanophenylethylamine (25CN-NBOH) (1.5 mg/kg, subcutaneous) on respiration rate of isoflurane-anaesthetised mice at (a) physiological and (b) elevated pad temperature. Mean ± SEM (-fold change of baseline brpm); *n* = 8–14 per group. Repeated measures analysis of variance (ANOVA) comparison to saline, ^*^p ⩽ 0.05, ^**^p ⩽ 0.01, and (NS) *p* ⩽ 0.1 (Trend). Vertical dotted lines indicate time of drug injection.

Isoflurane-anesthetised mice overall exhibited a tendency for a decrease of respiration rate over time (Figure 3; repeated measures ANOVA, significant main effect *time F*(2.72, 100.58) = 47.46, *p* ⩽ 0.001). There was a significant main effect *group* (*F*(1, 37) = 8.27, *p* = 0.007) and a significant *time × group interaction* (*F*(2.72, 100.58) = 3.21, *p* = 0.03). Following up on the *time × group* interaction, 25CN-NBOH significantly decreased the respiration rate at all four post-injection measurements of the 37°C condition (control vs. NBOH (mean ± SEM, fold change): 0.96 ± 0.041 vs. 0.88 ± 0.038 (5 min), *p* = 0.01; 0.91 ± 0.041 vs. 0.78 ± 0.053 (10 min), *p* = 0.014; 0.87 ± 0.048 vs. 0.72 ± 0.046 (15 min), *p* = 0.025; 0.87 ± 0.051 vs. 0.7 ± 0.048 (20 min), *p* = 0.01), but only as a trend at the 5-minute post-injection measurement of the 41°C condition (0.94 ± 0.015 vs. 0.86 ± 0.025 (5 min), *p* = 0.073). Mean absolute values for the pre- versus post-injection intervals (given in brpm) are listed in Tables 1 and 2.

### Oxygenation

**Figure 4.**
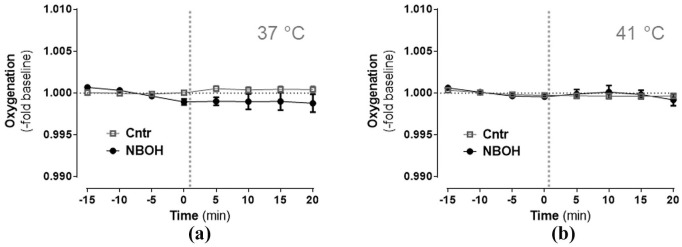
Effect of N-(2-hydroxybenzyl)-2,5-dimethoxy-4-cyanophenylethylamine (25CN-NBOH) (1.5 mg/kg, subcutaneous) on blood oxygenation of isoflurane-anaesthetised mice at (a) physiological and (b) elevated pad temperatures. Mean ± SEM (fold change of baseline % oxygen saturation); *n* = 8–14 per group. Repeated measures analysis of variance (ANOVA) comparison to saline, not significant. Vertical dotted lines indicate time of drug injection.

Overall, there was a slight tendency for blood oxygenation to decrease during anaesthesia, as implied by a significant main effect *time* in the repeated measures ANOVA (*F*(1.81, 66.96) = 3.24, *p* = 0.05) (Figure 4). Main effect *group* and *time* × *group* (× *temperature*) interactions turned out insignificant, indicating that blood oxygenation was not affected by 25CN-NBOH or its interaction with the pad temperature. Mean absolute values for the pre- versus post-injection intervals (given in % saturation) are presented in Tables 1 and 2.

### Core body temperature

**Figure 5.**
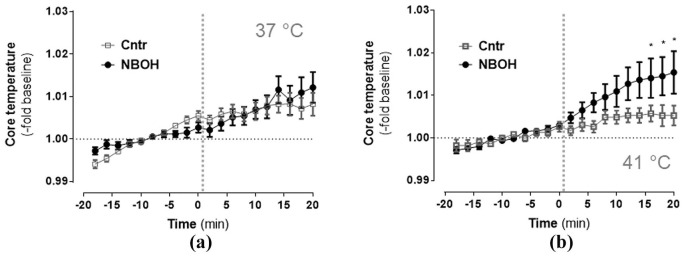
Effect of N-(2-hydroxybenzyl)-2,5-dimethoxy-4-cyanophenylethylamine (25CN-NBOH) (1.5 mg/kg, subcutaneous) on core body temperature of isoflurane-anaesthetised mice at (a) physiological and (b) elevated pad temperatures. Mean ± SEM (fold change of baseline °C); *n* = 6–9 per group. Repeated measures analysis of variance (ANOVA), post-hoc comparison to saline, ^*^p ⩽ 0.05. Vertical dotted lines indicate time of drug injection.

For a subpopulation of animals (*n* = 6–9 per group), the core body temperature was quantified via a rectal probe. Over the 40-minute observation period, the rectal temperature was noted to slightly increase across study groups (*time, F*(1, 25) = 36.53, *p* ⩽ 0.001). *Time × treatment* interaction likewise was significant (*F* (1, 25) = 9.13, *p* = 0.006), with a marginal contribution from pad temperature (*time* × *group* × *temperature F*(1, 25) = 1.85, *p* = 0.095). Follow-up analysis revealed the *time* × *group* interaction was driven by 25-NBOH favouring body warming during the last intervals of recording at 42°C (control vs. NBOH (mean ± SEM, fold change): 1.006 ± 0.0019 vs. 1.014 ± 0.005 (16 min), 1.0053 ± 0.003 vs. 1.014 ± 0.005 (18 min), and 1.005 ± 0.002 vs. 1.02 ± 0.005 (20 min), each *p* ⩽ 0.05) (Figure 5). Mean absolute values for the pre- versus post-injection intervals (given in °C) can be extracted from Tables 1 and 2.

## Discussion

Serotonin secreted into the blood stream is a major tissue hormone with a variety of actions on the cardiovascular system and, when released from the brain stem’s raphe nuclei, also mediates the neuronal regulation of blood pressure (Côté et al., 2004; Watts et al., 2012). Assuming the serotonergic vasoactivity of psychedelics might contribute to their overall brain dynamics, here we used the selective 5-HT_2A_R agonist 25CN-NBOH and investigated its effect on the parameters of neck-arterial blood flow.

At the physiological pad temperature, 25CN-NBOH induced a slight increase in neck-arterial pulse distention, a measure of local tissue perfusion that parallels changes in mean arterial blood pressure (Hete et al., 2013; Olivera et al., 2010). Pulse oximetry is largely driven by strong-pulse arteries. Using the most dominant neck arteries with immediate pulse from the heart-imminent aortic arch (Ruberte et al., 2017), our collar clip-based pulse distention measurement most likely reflects haemodynamics of the carotid arteries. The effect of 25CN-NBOH on neck-arterial blood flow is in line with diverse reports on psychedelics increasing blood pressure (Dedeoğlu and Fisher, 1991; Dolder et al., 2017; Huang and Ho, 1972; Wolbach et al., 1962), possibly resulting from increased total peripheral resistance (Chaouche-Teyara et al., 1994) due to vasoconstriction (Cohen, 1988). The rise in arterial distension was more pronounced when 25CN-NBOH was applied at the elevated pad temperature. Similarly, the 25CN-NBOH-induced rise in body temperature only occurred at 41°C, not at 37°C. Psychedelics evoke hyperthermia (e.g. Jacob and Lafille, 1963), particularly when primed by environmental heat load (Buchborn et al., 2016). Both the effects on core body temperature and on pulse distention were temperature dependent, which suggests a close relationship. Indeed, psychedelic-induced hyperthermia (like hypertension) is thought to engage 5-HT_2A_R-related vasoconstriction in the cutaneous bed, which interferes with heat dissipation (Blessing and Seaman, 2003). Increased body temperatures might affect how common carotid blood flow is distributed across external versus internal branches in relation to the brain’s thermoregulatory needs (Baker et al., 1982). Furthermore, 25CN-NBOH evoked tachycardia, which at the *physiological* pad temperature sustained but quickly reversed at the elevated pad temperature. Mirroring this observation, psychedelics have been demonstrated to increase heart rate both in humans (Dolder et al., 2017; Strassman and Qualls, 1994; Wolbach et al., 1962) and anaesthetised animals (Anderson et al., 1995; Friedman et al., 1978; Knowles and Ramage, 1999); for animals, results appear more controversial with several reports of bradycardia (Chaouche-Teyara et al., 1994; Tadepalli et al., 1975), inconsistencies or a lack of effects (Dabiré et al., 1989; Dedeoğlu and Fisher, 1991). The controversy in literature might partially arise from the possible adrenergic pharmacodynamics of the *earlier-generation* selective 5-HT_2_R agonists used (Ray, 2010). Indeed, bradycardia and the carotid-pressor effect of DOM show sensitivity to adrenergic antagonism (Huang and Ho, 1972; Tadepalli et al., 1975). Although there is other evidence pointing to a potential interaction of DOM and DOI with the adrenergic system (e.g. Alper, 1990; Buckholtz et al., 1988; Koskinen et al., 2003), 5-HT_2_R-comparable binding to adrenergic receptors has also been questioned (Leysen et al., 1989; Pierce and Peroutka, 1989). Adrenergic effects might alternatively be secondary to 5-HT_2_R-related adrenaline secretion from adrenal glands (Saxena and Villalón, 1990); thus, additional research, preferentially using functional assays, is needed to solidly clarify these issues. Beyond unselectivity, however, conflicting results on animal heart rates might also be reconciled for two opponent counterforces downstream of 5-HT_2A_Rs. Thus, tachycardia might be due to 5-HT_2A_R-mediated sympathetic cardiac nerve output (McCall and Harris, 1988; Anderson et al., 1995). Bradycardia, by contrast, might result from 5-HT_2A_R-mediated hypertension inviting the baroreceptor reflex to cancel out the sympathetically driven chronotropy (N’Diaye et al., 2001; Ramage et al., 1993). The tachycardic effect of 25CN-NBOH at the increased pad temperature was quickly reversed as pulse distention rose, which is in line with the proposed opponency. Apart from its effect on pulse distention and heart rate, 25CN-NBOH facilitated the development of bradypnea, which mirrors findings for DOI (Cayetanot et al., 2001; Khater-Boidin et al., 1999) and might be due to 5-HT_2_R-mediated inhibition of phrenic nerve activity (King and Holtman, 1990). Interestingly, phrenic inhibition induced by the psychedelic 5-MeO-DMT could be overcome by ambient heat (Lalley, 1982), which fits wells with the rapid reversal of 25CN-NBOH’s bradypneic effect at the increased pad temperature. Blood oxygen saturation was not significantly altered by 25CN-NBOH; the observed respiro-cardiovascular effects of the drug thus do not seem to reflect changes in functional arterial haemoglobin availability.

In summary, our results show that 25CN-NBOH induces a temperature-dependant increase in heart rate, a decrease in respiration rate and an increase in arterial blood flow, as measured by pulse oximetry drawn from brain-supplying (most likely carotid) arteries. At present, 25CN-NBOH is the most selective 5-HT_2_R agonist, with robust preference for 5-HT_2A_R over 5-HT_2B_ and 5-HT_2C_ receptors and lack of appreciable affinity for adrenergic receptors (Halberstadt et al., 2016; Hansen, 2010; Jensen et al., 2017). Given the primary relevance of 5-HT_2A_Rs for the psychedelic principle along with the immediacy of neck-arterial blood flow to the brain’s metabolic demands, our results might contribute to a more systemic understanding of how psychedelic-5-HT_2A_R interactions translate into the brain (haemo) dynamic characteristics for psychedelia.
